# Characterization and Discrimination of Key Aroma Compounds in Pre- and Postrigor Roasted Mutton by GC-O-MS, GC E-Nose and Aroma Recombination Experiments

**DOI:** 10.3390/foods10102387

**Published:** 2021-10-08

**Authors:** Huan Liu, Teng Hui, Fei Fang, Qianli Ma, Shaobo Li, Dequan Zhang, Zhenyu Wang

**Affiliations:** Key Laboratory of Agro-Products Processing, Institute of Food Science and Technology, Chinese Academy of Agricultural Sciences, Ministry of Agriculture and Rural Affairs, Beijing 100193, China; sd_lh1990@126.com (H.L.); htengui@163.com (T.H.); fangfeixdf@163.com (F.F.); mql201228@163.com (Q.M.); lishaobo@caas.cn (S.L.); dequan_zhang0118@126.com (D.Z.)

**Keywords:** roasted mutton, pre- and postrigor, key aroma compounds, marker, recombination and omission experiments

## Abstract

The key aroma compounds in the pre- and postrigor roasted mutton were studied in this study. The results showed that 33 and 30 odorants were detected in the pre- and postrigor roasted mutton, respectively. Eight aroma compounds, including 3-methylbutanal, pentanal, hexanal, heptanal, octanal, nonanal, 1-octen-3-ol, and 2-pentylfuran, were confirmed as key odorants by aroma recombination and omission experiments. The aroma profiles of pre- and postrigor roasted mutton both presented great fatty, roasty, meaty, grassy, and sweet odors. Particularly, the concentrations of hexanal, heptanal, octanal, nonanal, 1-octen-3-ol, and 2-pentylfuran in postrigor roasted mutton were significantly higher (*p* < 0.05) than those in the prerigor roasted mutton. The postrigor *back strap* was more suitable for roasting than the prerigor *back strap*. The pre- and postrigor roasted mutton could be obviously discriminated based on the aroma compounds by orthogonal partial least squares discrimination analysis (OPLS-DA) and principal component analysis (PCA). Hexanal and 1-octen-3-ol might potential markers for the discrimination of the pre- and postrigor roasted mutton.

## 1. Introduction

The production of mutton was 4.88 million tons in China in 2019, with a growth rate of 2.6%. Roasted mutton is the most popular meat product due to its unique aroma. However, a few studies have been reported to characterize the aroma compounds of roasted mutton after chilling at 4 °C for 72 h (postrigor), among which hexanal, octanal and nonanal are the main odorants according to odor activity values (OAVs) and concentrations based on an internal standard [1,2]. The majority of consumers prefer roasted mutton without chilling (prerigor) rather than those from chilled carcasses in most areas in China, and they believe that roasted mutton without chilling (prerigor) is more flavory than any chilled sample. However, the assumption is only a traditional point, and no scientific data deny it. To date, only one study about aroma compounds in the roasted mutton was reported at different aging times by the universal steam oven, among which higher peak areas of total aroma compounds were found in cooked mutton aged for 3 days than those aged for 1 day. However, the study did not elucidate key aroma compounds and aroma profiles in mutton by the traditional charcoal roasting process and did not clarify the aroma differences among the samples [2]. Our previous results showed that the shear force of roasted mutton aged for 1–24 h first increased (*p* < 0.05) and significantly decreased (*p* < 0.05) when aged for 1–7 days. Roasted mutton aged for 1 day had the highest shear force value. Sheep muscles aged for 1–12 h, 1 day and 3–7 days were considered to be in the prerigor, rigor and postrigor phases based on shear force and pH values, respectively [2]. The determination of key aroma compounds can provide data support for the selection of raw meat, the slaughter process, and cooking method optimization [3]. However, the aroma compounds in the roasted mutton are unclear. In particular, the data on the differences in key aroma compounds in the pre- and postrigor roasted mutton are rather scarce.

Recently, the sensomics approach has been widely applied in the characterization of key aroma compounds in samples [4,5]. Key aroma compounds could not be determined by the concentrations and OAVs of odorants alone. The OAV of decanal was higher than 1 in Beijing Youji broth (19) and commercial Broiler broth (2), whereas this odorant did not significantly affect the aroma profile of chicken broth, indicating that it was not the key aroma compound [3]. In contrast, aroma recombination experiment has recently been applied to determine the key aroma compounds in various foods, such as wine [4]. GC-MS can accurately identify, quantitate and determine key aroma compounds in samples. However, the mass spectrometry of GC-MS cannot be first translated into sensory perception response and second visually present the difference of samples. Interestingly, “E-sensing” technologies can clarify the overall aroma difference by simulating the human sense of nose, including e-nose [6]. The flash GC e-nose was a combination of GC and e-nose, which could effectively separate compounds and identify differences, such as virgin olive oils [7]. In particular, the integration of e-nose and GC-MS could comprehensively elucidate the aroma difference in samples, such as roasted bread, heated oil and virgin olive oil [8,9,10].

This study aimed to confirm the key aroma compounds and their differences in the pre- and postrigor roasted mutton. (i) The key aroma compounds were accurately identified and quantitated by gas chromatography olfactometry mass spectrometry (GC-O-MS). Afterward, (ii) the key aroma compounds in samples were determined by OAVs, contribution rates, and recombination and omission experiments. Then, (iii) it was confirmed that the postrigor *back strap* was more suitable for roasting than the prerigor *back strap*. Finally, (iv) the potential markers discriminating the pre- and postrigor roasted mutton were determined by GC-O-MS, flash GC e-nose, orthogonal partial least squares discrimination analysis (OPLS-DA), and principal component analysis (PCA).

## 2. Materials and Methods

### 2.1. Chemicals and Reagents

Standards of most volatile compounds were obtained from Sigma-Aldrich (Shanghai, China): 1-octen-3-ol (98%), (*E*)-2-octen-1-ol (97%), 1-heptanol (98%), propanal (97%), pentanal (98%), hexanal (98%), heptanal (97%), octanal (99%), (*E*)-2-octenal (97%), nonanal (99.5%), (*E*)-2-nonenal (97%), benzaldehyde (99.5%), 2-pentylfuran (98%), and 2,3-pentanedione (97%). The 3-methylbutanal (98%) was supplied by Aladdin (Shanghai, China). The n-alkanes (C_7_-C_40_, ≥97%, external standard) was obtained from o2si Smart Solutions (Shanghai, China). The 2-methyl-3-heptanone (99%) was supplied by Dr. Ehrenstorfer (Beijing, China) as an internal standard.

### 2.2. Sample Preparation

All animal procedures performed in this study were approved by the Animal Care and Use Committee of the Institute of Food Science and Technology, Chinese Academy of Agricultural Sciences (Beijing, China). A total of 120 sheep (6-month-old, small-tail sheep × Mongolian sheep with 27.40 ± 2.64 kg carcass weight) were pastured together in Inner Mongolia Province in China. All sheep had the same genetic background and were fed the same diet. 12 Sheep were randomly selected from 120 sheep. The *back strap* was obtained according to the cutting technical specification of mutton, which was same with *backstrap 5101* in the seventh edition of Handbook of Australian Meat [11]. The pre- and postrigor muscles were applied in each carcass. The left carcass was treated with prerigor, and the right half was treated with postrigor in each carcass. The prerigor *back straps* were the muscles from 12 carcasses (pH: 6.42 ± 0.08), which were quickly frozen at −35 °C within 45 min after slaughter. The postrigor *back straps* were the muscles from the 12 carcasses (pH: 5.42 ± 0.27), which were kept at 4 °C for 72 h and thereafter frozen at −35 °C. All samples were wrapped with nylon/polyethylene, transported to our lab by cold-chain logistics and stored at −80 °C. The muscles were incubated (MIR-154-PC, Panasonic, Japan) at 4 ± 1 °C overnight thaw until the core temperatures dropped to the range of −3 and −5 °C. After being trimmed off connective tissue and surface fat, the samples were cut into cubes (3 × 1.5 × 1.5 cm^3^). The samples were roasted for 10 min by traditional burning charcoal. The roasting process ended when the core temperature reached 77.6–79.9 °C in the samples (surface temperature: 85–97 °C).

### 2.3. GC-O-MS Analysis

Aroma compounds were extracted by the headspace solid-phase microextraction (HS-SPME) with a carboxen−polydimethylsiloxane fused silica (CAR/PDMS, 75 μm) coating fiber [12]. The minced sample and 2-methyl-3-heptanone (internal standard, 1.5 μL, 1.7 μg·μL^−1^) were put into a 20 mL vial sealed with a PTFE-silicon stopper. The vial was incubated at 55 °C for 10 min and the aroma compounds were extracted at 55 °C for 45 min. Immediately, the coating fiber was desorbed at 250 °C for 3 min. The analysis was prepared on an Agilent gas chromatograph (7890B) coupled with an olfactometry (ODP C200, Gerstal, Mulheim an der Ruhr, Germany) and 5977A mass selective detector. The aroma compounds were separated by a fused-silica capillary column (60 m × 250 μm × 0.25 μm, DB-Wax capillary column, Agilent Technologies, Santa Clara, CA, USA). The temperature program of the GC oven was 40 °C for 3 min, raised to 70 °C at 2 °C/min, increased to 130 °C at 3 °C/min, ramped to 230 °C at 10 °C/min and maintained for 10 min. The helium (99.99%) was prepared as a carrier gas with a flow rate of 1.4 mL/min. The injector temperature was kept at 250 °C with a splitless inlet. The electron ionization mode was positive ion (70 eV) with an acquisition range from 40 to 500 *m/z* in full-scan mode.

### 2.4. Identification Analysis of Aroma Compounds

Aroma compounds were identified by mass spectrometry library, linear retention indices (LRIs), odor qualities, and authentic flavor standards. LRIs were obtained according the retention time of n-alkanes (C_7_–C_40_) by linear interpolation. The aroma compounds were also determined by professional panelists using GC-O. Meanwhile, the authentic standards of aroma compounds were analyzed with the same detection procedure as that used for the samples. The aroma compounds were confirmed by retention times between authentic flavor standards and samples.

### 2.5. Quantitation Analysis of Aroma Compounds

Aroma compounds were quantitated by calibration curves of authentic flavor standards following semiquantitation of an internal standard. First, the concentrations of aroma compounds in the samples were evaluated according to the ratio of the concentration and peak area of the internal standard. In particular, the aroma compound concentrations with OAVs greater than 1 were calibrated by a 5-point standard curve of authentic flavor standards. Prior to quantitation analysis, the roasted mutton was prepared to obtain an artificial odorless matrix based on previous studies [12]. The calibration curves of aroma compounds in the roasted mutton were constructed by the above odorless matrix and authentic flavor standards with different concentrations. 2-Methyl-3-heptanone was put into the mixture to calibrate the peak area of aroma compounds. The odorless matrix without flavor standards was considered the control. Authentic flavor standards were analyzed by GC-SIM with the same detection procedure as that used for the samples. Authentic flavor standards, scanned ions (*m*/*z*) and calibration equations were obtained. The calibration curves of aroma compounds all have great linear correlations, where x is the ratio of the concentration of aroma compound to the internal standard and y is their peak ratio.

### 2.6. OAVs and Contribution Rate Analysis

The OAVs of aroma compounds were determined by dividing concentrations with their threshold [13]. The contribution rate was the OAV ratio of single aroma compound to total aroma compounds.

### 2.7. Aroma Recombination and Omission Experiments

The recombination and omission experiments were performed by a triangle test of sensory evaluation in a climate-controlled (26 ± 1 °C) sensory room [3]. A total of 50 sensory panelists aged 24–49 years old were screened and selected based on GBT 16291.1–2012. The panelists were trained for flavor recognition based on ISO 4121:2003. All panelists had been trained weekly and could describe and recognize odor qualities. Flavor profiles were determined using a scale from 0 to 5, which represented not detectable (0), very weak (1), weak (2), moderate (3), strong (4) and very strong (5) odors, respectively. The recombination model (model 1) was constructed by the above odorless matrix and authentic flavor standards with OAVs greater than 1. The sensory panelists evaluated the aroma similarity between model 1 and roasted mutton by a triangle test. Afterwards, the omission model (model 2) was prepared by omitting one aroma compound from model 1. The panelists estimated the aroma difference between model 1 and model 2. Finally, the recombination model (model 3) was prepared by an odorless matrix and aroma compounds that significantly affected the aroma profile of the samples. The panelists evaluated the aroma similarity between model 3 and the samples.

### 2.8. Flash GC E-Nose Analysis of Aroma Profile

A Heracles II e-nose (Alpha M.O.S., Toulouse, France) equipped with MXT-5 and MXT-1701 flame ionization detectors (FIDs) was used for the analysis of aroma compounds and aroma differences. The samples were treated as reported by Melucci and co-workers [7]. Briefly, the sample was heated at 50 °C for 30 min. Then, 3000 μL of headspace gas was injected into the GC port at a speed of 125 μL/s. The column temperature was 50 °C, rose to 250 °C at 2 °C/s and was maintained for 10 s. The temperatures of the GC port and FID were 200 °C and 260 °C, respectively. The aroma compounds were identified by retention indices from MXT-5 and MXT-1701 columns and determined by comparison with GC-MS data. The aroma differences in the pre- and postrigor roasted mutton were determined by PCA.

### 2.9. Statistical Analysis

All analyses were conducted in 12 measurements. Comparisons among roasted mutton of different aging times were performed using independent-samples t-test. The statistical analysis of aroma compounds in the roasted mutton were conducted at a level of *p* < 0.05 with SPSS 19.0 software (IBM Corporation, Armonk, NY, USA). Origin 2017 software and SIMCA 14.1 were used to perform plotting figures.

## 3. Results

### 3.1. Identification and Quantitation of Aroma Compounds in the Roasted Mutton

As presented in Table 1, Table 2 and Table 3, 33 aroma compounds were identified by GC-O-MS, among which 33 and 30 compounds were detected in the pre- and postrigor roasted mutton, respectively. Butanoic acid, pentanoic acid and 2,6-dimethylpyrazine were only found in prerigor samples. The aldehydes (10) and alcohols (7) with maximum types were the major odorants in the samples (Table 3). 3-Methylbutanal, pentanal, hexanal, heptanal, octanal, nonanal, and 1-octen-3-ol might be important odorants based on their high odor qualities (O) from GC-O-MS. The characteristic ion fragments of aroma compounds were obtained according to the identification of authentic flavor standards (Table 2).

To better understand the contributions of odorants to the aroma profile in samples, quantitation analysis was performed (Table 2 and Table 3). The pre- and postrigor roasted mutton both contained 15 compounds (OAVs > 1), which were quantitated based on the standard calibration curves of 5 points (Table 2). The major aroma compounds in the samples were propanal (105.86–152.67 ng/g), pentanal (1398.14–1407.06 ng/g), 2,3-pentanedione (115.22–208.95 ng/g), hexanal (3218.71–4383.43 ng/g), heptanal (744.04–1294.82 ng/g), 1-pentanol (162.93–165.20 ng/g), octanal (264.68–506.82 ng/g), 2,5-octanedione (170.57–537.81 ng/g), nonanal (119.41–197.01 ng/g), and 1-octen-3-ol (219.01–498.46 ng/g). In particular, the concentration of only 3-methylbutanal of 15 aroma compounds (OAVs > 1) in the prerigor roasted mutton was significantly higher (*p* < 0.05) than that of postrigor mutton. The concentrations of the 13 key aroma compounds in the prerigor roasted mutton were significantly lower (*p* < 0.05) than postrigor mutton, except 3-methylbutanal and pentanal.

### 3.2. Determination of Key Aroma Compounds in the Roasted Mutton

As shown in Table 3, the pre- and postrigor roasted mutton both contained 15 aroma compounds with OAVs greater than 1, including propanal, 3-methylbutanal, pentanal, 2,3-pentanedione, hexanal, heptanal, 2-pentylfuran, octanal, (E)-2-octenal, nonanal, 1-octen-3-ol, 1-heptanol, benzaldehyde, (E)-2-nonenal, and (E)-2-octen-1-ol. This result was also in agreement with the analysis of odor qualities. Among them, the highest OAVs were determined for hexanal (715.27-974.10), followed by 3-methylbutanal (243.96–426.56), octanal (378.12–724.02), 1-octen-3-ol (219.01–498.46), heptanal (248.02–431.61), and nonanal (119.41–197.01). The OAV of only 3-methylbutanal of the 15 aroma compounds in the prerigor roasted mutton was greater (*p* < 0.05) than that of postrigor mutton. The 13 aroma compounds had the reverse trends (*p* < 0.05). The changes in the contribution rates of aroma compounds were in accordance with those of OAVs, among which hexanal (29.92–31.62%) presented the highest contribution rate, followed by octanal (16.31–22.17%), 3-methylbutanal (7.40–18.85%), 1-octen-3-ol (9.57–15.32%), and heptanal (10.88–13.21%). These results preliminarily indicated that the 15 aroma compounds (OAVs > 1) with high contribution rates might be key odorants from the difference of aroma profiles in pre- and postrigor samples.

### 3.3. Confirmation of Key Aroma Compounds in the Roasted Mutton

**Figure 1:**
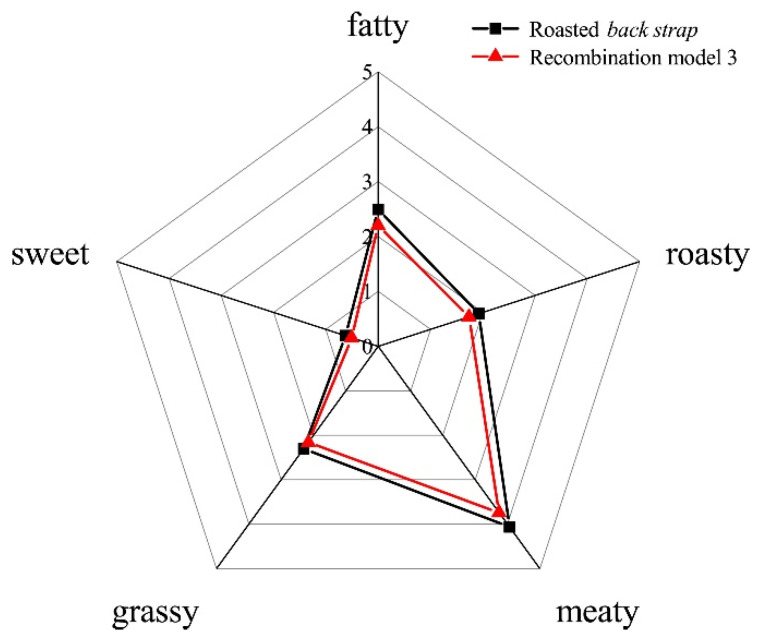
Aroma profiles of roasted mutton compared with the aroma recombination model 3.

The odorless matrix was constructed with 74.67% ultrapure water and authentic flavor standards (OAVs > 1) in the samples. The recombination model (model 1, 15 odorants) with all aroma compounds with OAVs greater than 1 revealed an extremely high similarity with the original roasted mutton in terms of the aroma profile by the triangle test. The results of the omission experiments (model 2, 14 odorants) indicated that 8 odorants significantly affected the overall aroma profile (*p* < 0.05) of the samples, including 3-methylbutanal, pentanal, hexanal, heptanal, 2-pentylfuran, octanal, nonanal, and 1-octen-3-ol. In particular, the model without hexanal and 1-octen-3-ol presented a noticeable difference (*p* < 0.01) compared to the aroma profile in model 1. Finally, the recombination model with the 8 aroma compounds mentioned above (model 3) showed a high similarity (4.51 out of 5 points) in comparison with roasted mutton, as illustrated in Figure 1. In particular, the pre- and postrigor roasted mutton both had fatty, roasty, meaty, grassy, and sweet odors. The intensity of the aroma profile in the postrigor roasted mutton was significantly greater (*p* < 0.05) than prerigor sample.

### 3.4. Potential Markers Analysis for Discriminating the Pre- and Postrigor Roasted Mutton Based on Aroma Compounds

**Figure 2:**
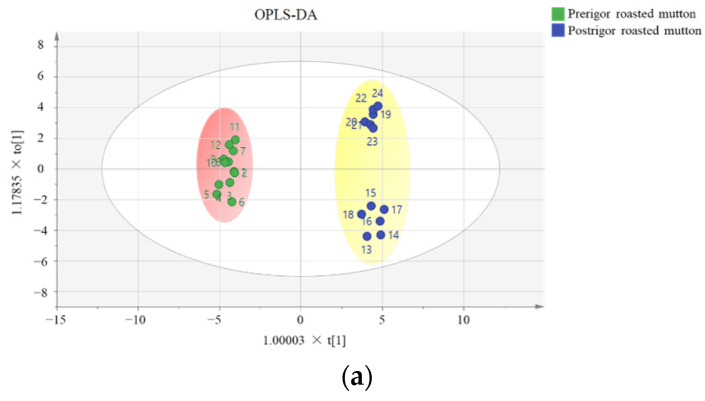

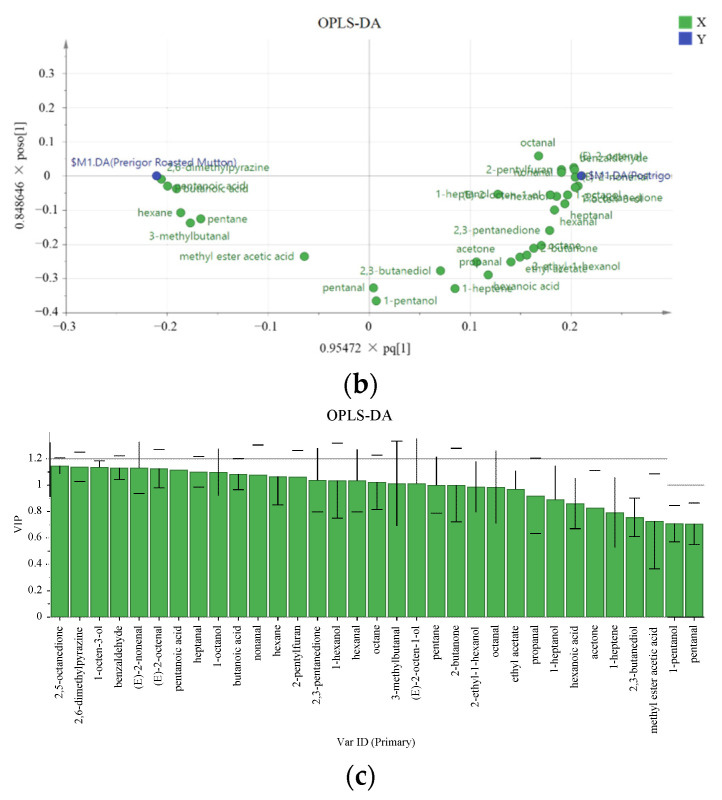
OPLS-DA of aroma compounds in the pre- and postrigor roasted mutton. (**a**) score scatter plot. (**b**) loading scatter plot. (**c**) VIP plot.

As presented in the score scatter plot of OPLS-DA (R^2^X = 0.92, R^2^Y = 0.99, Q^2^ = 0.99) (Figure 2), the pre- and postrigor roasted mutton were obviously separated. R^2^ and Q^2^ revealed the fitness and predictive ability of the model, respectively. The prerigor roasted mutton was in the second and third quadrants, in which aldehydes, acids, esters, alkanes, and nitrogen-containing compounds were the predominant chemical families, such as 3-methylbutanal, pentanoic acid, and 2,6-dimethylpyrazine. The postrigor roasted mutton was located in the first and fourth quadrants of the model, among which alcohols, aldehydes, ketones, and furans had an important contribution, including 1-octen-3-ol, hexanal, 2,3-pentanedione, and 2-pentylfuran. The aroma compounds (variable importance for the projection ≥ 1) were generally considered as potential markers to discriminate samples. A total of 20 aroma compounds were identified to show differences between pre- and postrigor mutton (Figure 2c), such as 2,5-octanedione, 2,6-dimethylpyrazine, 1-octen-3-ol, and hexanal. The results also indicated that the postrigor roasted mutton had richer aroma compounds than the prerigor roasted mutton.

**Figure 3:**
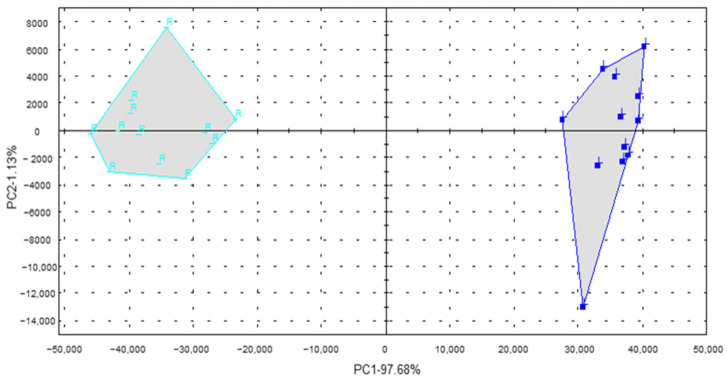
PCA of aroma compounds in the pre- and postrigor roasted mutton by flash GC e-nose. R and L represented the prerigor roasted mutton and postrigor roasted mutton, respectively.

To further quickly determine the differential aroma compounds in the pre- and postrigor roasted mutton, flash GC e-nose and PCA were used. As illustrated in Table 4, 11 aroma compounds were identified in the two samples by flash GC e-nose. Among these, hexanal had the maximum peak area, followed by pentanal, 1-octen-3-ol, and heptanal. In particular, the peak areas of most aroma compounds, including hexanal and 1-octen-3-ol, in the postrigor samples was significantly greater (*p* < 0.05) than that in the prerigor samples, which was consistent with the GC-O-MS results. PCA of the flash GC e-nose was performed to determine the correlation pattern with individual composition variables in the discrimination between the two samples. As presented in Figure 3, the accumulative variance contribution rate of the first two PCs was 98.81% higher than 85% (PC1 of 97.68% and PC2 of 1.13%), which was sufficient to discriminate between these two samples. The general aroma feature could be well distinguished by a flash GC e-nose coupled with PCA. Based on above analysis, hexanal and 1-octen-3-ol might be potential markers to discriminate the pre- and postrigor roasted mutton.

## 4. Discussion

### 4.1. Aldehydes and Alcohols Were Key Aroma Compounds in the Pre- and Postrigor Roasted Mutton

It was reported that aldehydes and alcohols were the most important aroma compounds in roasted meat [14]. It was clearly observed that these compounds mainly contribute to the overall aroma of samples, such as hexanal (OAVs: 715.27–974.10) and 1-octen-3-ol (OAVs: 219.01–498.46). This result was in agreement with previous studies [1,2], which showed that hexanal had the most abundant concentration in roasted mutton, followed by 1-octen-3-ol, nonanal, and octanal. In particular, 8 of 15 odorants (OAVs > 1) comprising 6 aldehydes and 1 alcohol were confirmed as key odorants by the recombination and omission experiments. This result also corresponded to the studies, in which hexanal, heptanal, octanal, nonanal, and 1-octen-3-ol had the higher concentrations and OAVs in grilled goat meat [15]. The roasted mutton had strong roasty, fatty, grassy, meaty, and sweet odors, which were mainly caused by aldehydes and alcohols derived from the degradation of lipids and Strecker degradation of amino acids [16]. The phospholipids contained more unsaturated fatty acids than triacylglycerols, which caused the former’s predominant contributions to the formation of fatty aldehydes and alcohols [17]. Pentanal, hexanal, heptanal, and 1-octen-3-ol could be generated from the oxidation of unsaturated fatty acids, which were responsible for the grassy note [3,18,19,20]. Aldehydes containing octanal and nonanal might predominantly contribute to fatty aromas [12]. 3-Methylbutanal, a Strecker aldehyde, was detected in the Maillard reaction with a seasoning-like odor [21]. In addition, the ketones and alkylfurans, including 2,3-pentanedione and 2-pentylfuran, could also be generated from the decomposition of lipids, which could generate roasty and meaty notes, respectively [22,23,24]. In particular, the aroma profile of roasted mutton was formed by the synergistic effect of key odorants rather than a single component [25]. Meanwhile, the concentrations of most key aroma compounds in the postrigor roasted mutton were significantly higher than those of the prerigor mutton, such as hexanal, heptanal, octanal, nonanal, 1-octen-3-ol, and 2-pentylfuran. This result indicated the postrigor *back strap* was more suitable for roasting than the prerigor *back strap*. This phenomenon was also consistent with the study reported by Coppock and Macleod, who clarified that the aging time generated more aroma compounds in the boiled beef [26]. Both thermal oxidation and autoxidation could produce the aldehydes and alcohols in meat and meat products. The richer aroma compounds in the postrigor roasted mutton could be explained by the autoxidation during aging [27,28].

### 4.2. Pre- and Postrigor Roasted Mutton were Discriminated Based on Key Aroma Compounds by GC-O-MS and GC E-Nose

In this study, GC-MS provided reliable and comprehensive diagnostic information for the detection of 8 key compounds, among which the concentration differences of 8 key odorants were responsible for the discrimination of the overall aroma profile of pre- and postrigor roasted mutton. In particular, hexanal and 1-octen-3-ol predominantly contributed to the aroma profile and caused the aroma difference of samples by using GC-O-MS. Meanwhile, the aroma profiles were obviously separated in the pre- and postrigor roasted mutton by using flash GC-O-MS, GC e-nose, OPLS-DA, and PCA, which was in agreement with aroma analysis of other food [29]. The characterization and discrimination of aroma compounds in the pre- and postrigor roasted mutton could also be successfully identified by GC-O-MS, among which hexanal and 1-octen-3-ol were key odorants and resulted in the difference of aroma profile in samples. The flash GC e-nose performance in the discrimination was consistent with respect to GC-O-MS, which was identical to previous studies [8,28]. These results indicated that hexanal and 1-octen-3-ol might be potential markers for discriminating the pre- and postrigor roasted mutton. This was in accordance with previous studies, among which hexanal and 1-octen-3-ol were indicators of oxidative stability and flavor acceptability in foods [30,31]. In addition, the combination of GC-MS with an e-nose could provide a comprehensive analysis for the characterization and discrimination of aroma compounds.

## 5. Conclusions

In this study, a total of 33 and 30 odorants were identified in the pre- and postrigor roasted mutton, among which they belonged to 8 chemical classes, such as aldehydes, ketones, alcohols, furans, acids, esters, and nitrogen-containing compounds. Eight odorants were confirmed to be the key aroma compounds in the roasted mutton, including hexanal, octanal, 1-octen-3-ol, nonanal, heptanal, pentanal, 3-methylbutanal, and 2-pentylfuran. The sensory evaluation of the recombination model including 8 key aroma compounds scored 4.51 out of 5 points. Only the concentration of 3-methylbutanal of 8 key aroma compounds in the prerigor roasted mutton was significantly higher than that of the postrigor mutton. Other 6 key aroma compounds, including hexanal, octanal, 1-octen-3-ol, nonanal, heptanal, and 2-pentylfuran, had the reverse trends. The pre- and postrigor roasted mutton could be discriminated based on the aroma compounds by GC-O-MS, flash GC e-nose, OPLS-DA, and PCA. Hexanal and 1-octen-3-ol might be potential markers to discriminate the pre- and postrigor roasted mutton. This study confirmed the key aroma compounds in the roasted mutton. Most importantly, this study provided the scientific data to clarify that the postrigor *back strap* was more suitable for roasting.

## Figures and Tables

**Table 1:** Aroma compounds, linear retention indices (LRIs), and identification methods in the pre- and postrigor roasted mutton.

Compounds ^a^	LRIs		Identification ^d^
	Literature ^b^	Calculated ^c^	
Pentane	– ^e^	–	MS
Hexane	–	–	MS, S
1-Heptene	750	757	MS, LRI
Propanal	798	795	MS, LRI, O, S
Octane	–	–	MS, S
Acetone	814	816	MS, LRI
Methyl Ester Acetic Acid	827	827	MS, LRI
Ethyl Acetate	887	890	MS, LRI
2-Butanone	900	905	MS, LRI
3-Methylbutanal	915	918	MS, LRI, O, S
Pentanal	979	980	MS, LRI, O, S
2,3-Pentanedione	1060	1059	MS, LRI, O, S
Hexanal	1094	1088	MS, LRI, O, S
Heptanal	1188	1188	MS, LRI, O, S
2-Pentylfuran	1230	1234	MS, LRI, O, S
1-Pentanol	1261	1259	MS, LRI
Octanal	1291	1293	MS, LRI, O, S
2,5-Octanedione	–	1329	MS
2,6-Dimethylpyrazine	1338	1337	MS, LRI
1-Hexanol	1359	1362	MS, LRI
Nonanal	1396	1398	MS, LRI, O, S
(*E*)-2-Octenal	1434	1437	MS, LRI, O, S
1-Octen-3-Ol	1456	1458	MS, LRI, O, S
1-Heptanol	1462	1464	MS, LRI, O, S
2-Ethyl-1-Hexanol	1499	1497	MS, LRI
Benzaldehyde	1534	1537	MS, LRI, O, S
(*E*)-2-Nonenal	1549	1550	MS, LRI, O, S
1-Octanol	1573	1571	MS, LRI
2,3-Butanediol	1583	1589	MS, LRI
(*E*)-2-Octen-1-Ol	1622	1624	MS, LRI, O, S
Butanoic Acid	1644	1642	MS, LRI
Pentanoic Acid	1720	1724	MS, LRI
Hexanoic Acid	1854	1856	MS, LRI

^a^ The aroma compounds in the pre- and postrigor roasted mutton. ^b^ Reported data in literatures. ^c^ Data calculated based on the retention time of n-alkanes (C_7_–C_40_) by linear interpolation. ^d^ Identified methods. MS, mass spectrometry library of GC-MS; LRI, linear retention indices; O, odor qualities; S, authentic flavor standards. ^e^ Not found or calculated.

**Table 2:** Ion fragments and standard calibration curves of aroma compounds (OAVs >1) in the pre- and postrigor roasted mutton.

Compounds	Ion Fragments ^a^	Standard Calibration Curves ^b^	R^2^
Propanal	27, 28, 29, 58	y = 0.0001x + 0.0022	0.995
3-Methylbutanal	41,43,44, 58	y = 0.00004x + 0.0009	0.990
Pentanal	29, 41, 44, 58	y = 0.0002x + 0.0016	0.990
2,3-Pentanedione	27, 29, 43, 57	y = 0.0001x − 0.0002	0.987
Hexanal	41, 44, 56, 57	y = 0.0008x + 0.1074	0.989
Heptanal	41, 43, 44, 70	y = 0.0003x + 0.0021	0.999
2-Pentylfuran	53, 81, 82, 138	y = 0.004x + 0.0012	0.998
Octanal	41,43, 56, 84	y = 0.0002x + 0.018	0.994
Nonanal	41, 43, 56, 57	y = 0.0011x + 0.0023	0.997
(*E*)-2-Octenal	29, 41, 55, 70	y = 0.0004x + 0.0007	0.988
1-Octen-3-Ol	43, 55, 57, 72	y = 0.0004x + 0.0086	0.999
1-Heptanol	41, 55, 56, 70	y = 0.0019x − 0.0071	0.997
Benzaldehyde	51, 77, 105, 106	y = 0.0067x − 0.0265	0.994
(*E*)-2-Nonenal	41, 43, 55, 70	y = 0.0105x − 0.0097	0.992
(*E*)-2-Octen-1-Ol	41, 43, 55, 57	y = 0.0011x − 0.0038	0.999

^a^ Selected ion fragments based on the authentic flavor standards. ^b^ Equations of standard calibration curves, where x is the concentration ratio of authentic flavor standards to internal standard and y is the peak area ratio of authentic flavor standards to internal standard. The pre- and postrigor roasted mutton both contained the 15 aroma compounds.

**Table 3:** Concentrations, OAVs, and contribution rates of aroma compounds in the pre- and postrigor roasted mutton.

Compounds	Concentration (ng/g) ^a^		OAVs ^b^		Contribution Rates ^c^	
	Pre-Rigor	Post-Rigor	Pre-Rigor	Post-Rigor	Pre-Rigor	Post-Rigor
Pentane	21.38 ± 1.68 ^a^	10.13 ± 0.68 ^b^	0	0	0	0
Hexane	13.78 ± 0.77 ^a^	5.85 ± 0.38 ^b^	0	0	0	0
1-Heptene	3.80 ± 0.15 ^b^	4.42 ± 0.25 ^a^	0	0	0	0
Propanal	105.86 ± 2.99 ^b^	152.67 ± 10.72 ^a^	11.14 ± 0.31 ^b^	16.07 ± 1.13 ^a^	0.49 ± 0.02	0.49 ± 0.03
Octane	14.69 ± 0.50 ^b^	21.45 ± 0.89 ^a^	0	0	0	0
Acetone	13.33 ± 0.71 ^b^	16.00 ± 0.71^a^	0	0	0	0
Methyl Ester Acetic Acid	8.72 ± 0.58	7.62 ± 0.42	0	0	0	0
Ethyl Acetate	9.46 ± 0.60 ^b^	15.32 ± 1.08 ^a^	0.10 ± 0.01 ^b^	0.15 ± 0.01 ^a^	0	0.01 ± 0.00
2-Butanone	4.93 ± 0.26 ^b^	8.71 ± 0.59 ^a^	0	0	0	0
3-Methylbutanal	85.31 ± 1.94 ^a^	48.79 ± 4.97 ^b^	426.56 ± 9.71 ^a^	243.96 ± 24.84 ^b^	18.85 ± 0.63 ^a^	7.40 ± 0.68 ^b^
Pentanal	1398.14 ± 33.58	1407.06 ± 81.28	116.51 ± 2.80	117.26 ± 6.77	5.12 ± 0.08 ^a^	3.58 ± 0.17 ^b^
2,3-Pentanedione	115.22 ± 3.01 ^b^	208.95 ± 11.61 ^a^	5.76 ± 0.15 ^b^	10.45 ± 0.58 ^a^	0.26 ± 0.01 ^b^	0.32 ± 0.02 ^a^
Hexanal	3218.71 ± 75.44 ^b^	4383.43 ± 114.32 ^a^	715.27 ± 16.77 ^b^	974.1 ± 25.40 ^a^	31.62 ± 1.10	29.92 ± 0.66
Heptanal	744.04 ± 23.91 ^b^	1294.82 ± 44.14 ^a^	248.02 ± 7.97 ^b^	431.61 ± 14.71 ^a^	10.88 ± 0.17 ^b^	13.21 ± 0.24 ^a^
2-Pentylfuran	13.26 ± 0.36 ^b^	20.91 ± 0.73 ^a^	2.21 ± 0.06 ^b^	3.49 ± 0.12 ^a^	0.10 ± 0.00 ^b^	0.11 ± 0.00 ^a^
1-Pentanol	162.93 ± 6.09	165.20 ± 9.19	0.04 ± 0.00	0.04 ± 0.00	0	0
Octanal	264.68 ± 27.51 ^b^	506.82 ± 28.84 ^a^	378.12 ± 39.31 ^b^	724.02 ± 41.20 ^a^	16.31 ± 1.36 ^b^	22.17 ± 1.09 ^a^
2,5-Octanedione	170.57 ± 8.26 ^b^	537.81 ± 9.87 ^a^	0	0	0	0
2,6-Dimethylpyrazine	11.07 ± 0.45 ^a^	0 ^b^	0.03 ± 0.00 ^a^	0 ^b^	0	0
1-Hexanol	25.39 ± 1.79 ^b^	48.87 ± 2.02 ^a^	0.01 ± 0.00 ^b^	0.02 ± 0.00 ^a^	0	0
Nonanal	119.41 ± 4.51 ^b^	197.01 ± 6.38 ^a^	119.41 ± 4.51 ^b^	197.01 ± 6.38 ^a^	5.23 ± 0.13 ^b^	6.05 ± 0.18 ^a^
(*E*)-2-Octenal	8.86 ± 0.29 ^b^	21.52 ± 0.69 ^a^	2.96 ± 0.10 ^b^	7.17 ± 0.23 ^a^	0.13 ± 0.01 ^b^	0.22 ± 0.01 ^a^
1-Octen-3-Ol	219.01 ± 9.90 ^b^	498.46 ± 10.96 ^a^	219.01 ± 9.90 ^b^	498.46 ± 10.96 ^a^	9.57 ± 0.27 ^b^	15.32 ± 0.31 ^a^
1-Heptanol	15.26 ± 0.38 ^b^	17.00 ± 0.33 ^a^	5.09 ± 0.12 ^b^	5.67 ± 0.0.11 ^a^	0.22 ± 0.00 ^a^	0.18 ± 0.00 ^b^
2-Ethyl-1-Hexanol	1.76 ± 0.12 ^b^	3.25 ± 0.26 ^a^	0	0	0	0
Benzaldehyde	7.62 ± 0.04 ^b^	8.88 ± 0.05 ^a^	2.54 ± 0.01 ^b^	2.96 ± 0.02 ^a^	0.11 ± 0.00 ^a^	0.09 ± 0.00 ^b^
(*E*)-2-Nonenal	1.59 ± 0.00 ^b^	1.80 ± 0.01 ^a^	19.92 ± 0.04 ^b^	22.58 ± 0.14 ^a^	0.88 ± 0.02 ^a^	0.70 ± 0.02 ^b^
1-Octanol	12.24 ± 0.42 ^b^	21.77 ± 0.64 ^a^	0.11 ± 0.00 ^b^	0.20 ± 0.01 ^a^	0^b^	0.01 ± 0.00 ^a^
2,3-Butanediol	4.31 ± 0.57	5.65 ± 0.59	0	0	0	0
(*E*)-2-Octen-1-Ol	14.94 ± 0.39 ^b^	23.06 ± 0.93 ^a^	4.98 ± 0.13 ^b^	7.69 ± 0.31 ^a^	0.22 ± 0.00	0.24 ± 0.01
Butanoic Acid	1.06 ± 0.10 ^a^	0 ^b^	0	0	0	0
Pentanoic Acid	1.63 ± 0.11 ^a^	0 ^b^	0	0	0	0
Hexanoic Acid	27.29 ± 1.69 ^b^	50.54 ± 4.28 ^a^	0.01 ± 0.00 ^b^	0.02 ± 0.00 ^a^	0	0

^a^ Concentrations of aroma compounds were calculated according to standard calibration curves of 5 points. ^b^ OAVs were calculated by dividing concentrations with their threshold. ^c^ Contribution rates were the OAV rates of individual compound to all compounds.

**Table 4:** Peak areas of aroma compounds detected by flash GC e-nose in the pre- and postrigor roasted mutton.

Compounds (Peak Area)	Roasted Mutton	
	Pre-Rigor	Post-Rigor
Propanal	1039.59 ± 31.03	1036.75 ± 33.45
Hexane	489.23 ± 30.71	545.17 ± 49.23
3-Methylbutanal	251.64 ± 3.01 ^b^	273.25 ± 7.56 ^a^
Pentanal	7548.89 ± 108.17 ^b^	8558.67 ± 189.74 ^a^
2,3-Pentanedione	175.81 ± 19.61	177.83 ± 19.76
2,3-Butanediol	1875.48 ± 63.96	2063.67 ± 89.75
Hexanal	77261.81 ± 1382.88 ^b^	87650.17 ± 1309.27 ^a^
1-Hexanol	112.67 ± 2.40 ^b^	135.58 ± 4.32 ^a^
Heptanal	2079.92 ± 43.80 ^b^	2627.92 ± 132.13 ^a^
1-Octen-3-Ol	2498.33 ± 52.11 ^b^	2934.42 ± 129.93 ^a^
Octanal	614.85 ± 22.62 ^b^	712.08 ± 12.94 ^a^

Data with different superscripts (^a^,^b^) within each row indicate significant difference (*p* < 0.05).

## Data Availability

The data presented in this study are available on request from the corresponding author.
